# The mediating role of residents’ wellbeing between program leadership and quality of care: A cross-sectional study

**DOI:** 10.1371/journal.pone.0259800

**Published:** 2021-11-08

**Authors:** Fatima Msheik-El Khoury, Diana Dorothea Naser, Zin Htway, Salah Zein El Dine

**Affiliations:** 1 College of Health Professions, Walden University, Minneapolis, MN, United States of America; 2 Faculty of Medicine, American University of Beirut, Beirut, Lebanon; Shahrood University of Medical Sciences, ISLAMIC REPUBLIC OF IRAN

## Abstract

**Background:**

Research has shown that organizational leadership and support affect organizational outcomes in several sectors, including healthcare. However, less is known about how organizational leadership might influence the wellbeing of clinical trainees as well as the quality of their patient care practices.

**Objectives:**

This study examined the mediating effects of burnout and engagement between program director-resident relationship quality and residents’ reported quality of care, and the moderating effect of perceived departmental support.

**Methods:**

The authors conducted a cross-sectional study in September 2020, using a 41-item questionnaire, among 20 residency programs in an academic medical center in Lebanon. Measures included program director-resident relationship quality, perceived departmental support, burnout subcomponents, engagement, and self-reported quality of care. Ordinary least squares regression was used to conduct parallel mediation and moderated mediation analyses using SPSS macro-PROCESS, to assess the strength and direction of each of the proposed associations.

**Results:**

A total of 95/332 (28.6%) residents responded. Results revealed that program director-resident relationship quality had a significant indirect effect on residents’ suboptimal patient care practices and attitudes towards patients, through at least one of the wellbeing dimensions (*p* < .05). Perceived departmental support did not play a dominant role over program director-resident relationship quality, and thus did not influence any of the mediated relationships.

**Conclusion:**

Our study adds a new dimension to the body of literature suggesting that program director-resident relationship quality plays an important role in promoting residents’ wellbeing and achieving important clinical health outcomes. Such findings imply that the quality of program director-resident relationship could be an important component of residents’ wellbeing and patient safety. If further research confirms these associations, it will become imperative to determine what interventions might improve the quality of relationships between program directors and residents.

## Introduction

Resident physicians have a dual role in the healthcare system, whereby they act as learners and medical care professionals simultaneously. In the field of medicine, the wellbeing of residents has garnered national interest due to its influence on patient safety and quality of care. This manuscript focuses particularly on residents’ burnout (emotional exhaustion and depersonalization) and engagement as two relatively independent dimensions of wellbeing, rather than two opposite poles of the same bipolar dimension [1]. Indeed, a survey conducted amongst resident doctors in the Netherlands has shown that many residents fulfill the criteria for burnout, while they scored high on work engagement [2]. Burnout is a result of a health impairment process characterized by physical and mental drainage caused by chronic exhausting job demands (heavy workload, poorly designed jobs, etc.), while engagement represents a motivational process exerted by available job resources (e.g., supervisory support, performance feedback, task-related resources) [3].

Several concerns related to patient safety outcomes (self-perceived medical errors and suboptimal care) were associated with residents’ burnout, as delineated by two recent systematic reviews [4, 5]. While burnout was extensively assessed, few studies examined how residents’ engagement affects the quality of patient care [6, 7]. In Lebanon specifically, research studies have been limited to assessing the prevalence of depressive symptoms and severity of burnout among residents without further investigation of potential relationships between residents’ wellbeing, job resources, and clinical outcomes [8, 9].

Scarce information is available on job resources that may help residents cope with heavy job demands, reduce the risk of burning out, promote engagement, and maintain an adequate quality of patient care. Recent literature focused on developing resiliency programs in order to promote residents’ wellbeing [10–12]. Resiliency interventions aimed to improve residents’ intrinsic personal qualities and skills e.g. leadership, communication, listening and conflict resolution, in order to moderate their responses to their learning/work environment and subsequently their well-being. Although mindfulness strategies have sometimes proven effective, it is not alone sufficient to teach residents coping skills and to assess the impact of these resiliency programs on residents’ wellbeing. Residents’ wellbeing cannot be entirely explained by deficits in the intrinsic qualities of the resident alone, but rather should be considered a consequence of the interaction between the clinical learning/work environment and intrinsic factors [13]. The clinical learning and work environment comprises organizational leadership, program culture, relationships with faculty, meaning at work, efficiency and resources, and mistreatment at work [14, 15]. Hence, interventions promoting wellbeing should also focus on correcting causative extrinsic factors within the clinical learning and work environment. For instance, research studies in medical education did not attempt to understand the role that residency program director leadership support plays in relation to residents’ wellbeing and productivity. Currently, program director leadership has not been defined outside the notion of expert and informational leader in the Accreditation Council of Graduate Medical Education Common Program Requirements. The program director position, being a position of leadership by virtue, should not only organize and implement curricula and evaluate residents, but also support, engage and inspire them, to direct their behavior towards a common goal [16]. Hence, including direct program leadership support in studies assessing residents’ wellbeing and their performance is highly warranted. In addition, residents’ clinical learning and work environment comprises chairpersons and faculty members whose way of support (favorably or unfavorably) may influence residents’ perceptions about how the department values their contributions and cares about their wellbeing according to the organization support theory [17].

### Conceptual framework

**Fig 1:**
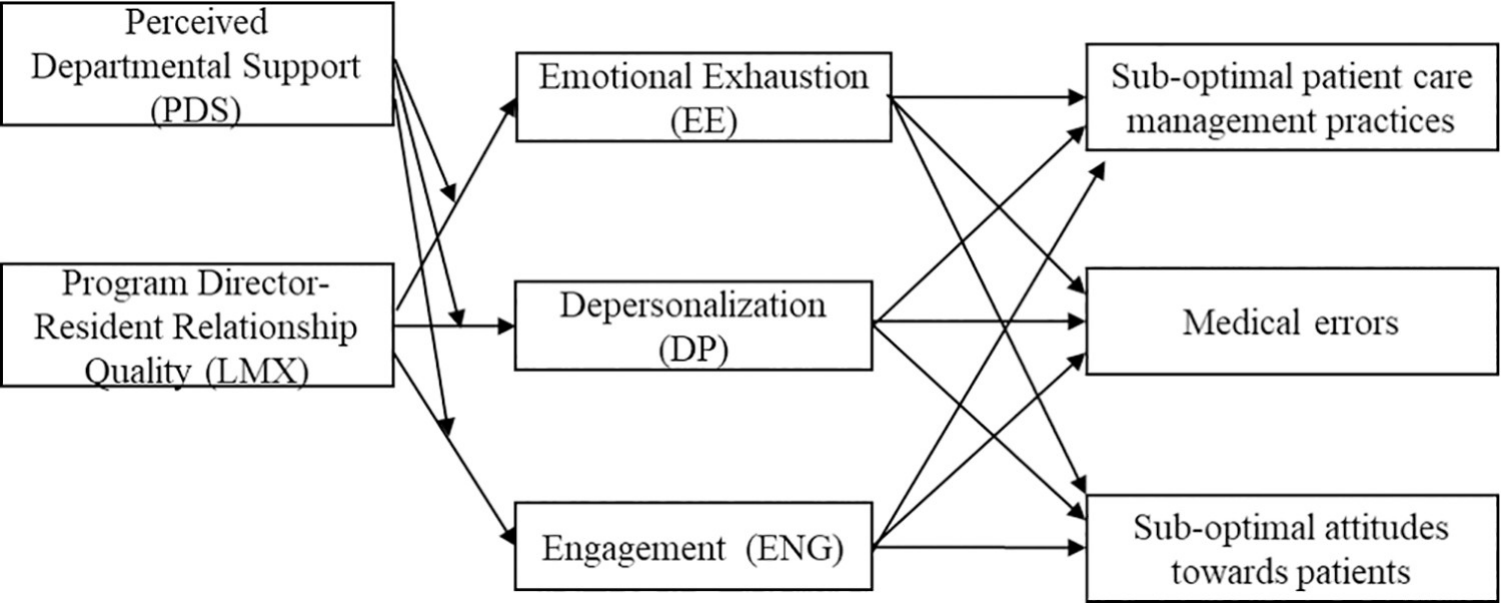
Conceptual framework for the study.

Several leadership and organizational theories have been studied in the literature such as transformational leadership theory, situational theory of leadership, and organizational culture theory, however, exchange-based constructs have been significantly used by organizational researchers for understanding workplace desired behavior and attitudes [18–21]. The leader-member exchange (LMX) and organizational support theories have been chosen to guide the study conceptual framework, shown in Fig 1. In addition to being antecedents to employee wellbeing and performance, these theories differ from other theories in that they place emphasis on the dyadic relationship between the leader and employee, as well as the employing organization and employee respectively [21].

In terms of a high-quality leader-member exchange relationship, the employee would feel obligated to perform the job adequately and engage in behaviors that directly benefit the leader and are beyond the scope of usual job expectations [22]. In terms of organizational support theory, reciprocity is different because the organization is made up of many individuals [22]. Hence, feelings of obligation and commitment towards the organization are based on a history of organizational decisions taken by immediate supervisors, higher supervisors, or individuals in the organization not part of the supervisory channel [23]. However, both theories state that a high-quality relationship with a supervisor or an organization is a resource for employees to help them alleviate job-associated stress and hence instill psychological safety [24]. In addition, a high-quality leader-member exchange relationship is positively related to feelings of energy, which, in turn, is related to greater work engagement among employees [25]. Ultimately, this promotes employees’ capacities to deal with job demands and work strain and improves their motivation, which enhances their performance [24–26]. Based on the above-mentioned theories, our conceptual framework model explains how the quality of the program director-resident relationship can act as a job resource that may indirectly influence residents’ performance in terms of quality of care through residents’ wellbeing. Studies have also shown that perceived organizational support is more predictive of psychological outcomes, while the leader-member exchange is more predictive of behavioral outcomes [27]. Since intent precedes behavior [28], the framework of this study also suggested that perceived departmental support is a necessary condition for residents in high-quality relationships with their program directors to be more engaged and experience lower levels of emotional exhaustion and depersonalization, and eventually perform well.

### Aim of the study

This study aimed to examine 1) the relationship between the program director-resident relationship quality and residents’ reported quality of care, 2) the mediating effects of burnout and engagement, and 3) the moderating effect of perceived departmental support.

## Methods

In September 2020, we conducted a cross-sectional study among residents from all specialties in in Lebanon. To avoid selection bias, we attempted to recruit resident physicians who were similar to each other and to the larger population of residents. Hence, we selected residents from the largest academic medical center in Lebanon. This center offers 20 residency training programs whereby residents are trained by a clinical teaching team, and each program is led by a program director. A total of 332 residents received an invitation email to participate in this survey, which was administered via online software (Lime Survey). The invitation was comprised of a brief synopsis of the study and its aims, along with an informed consent to be signed electronically. The informed consent included that participation was voluntary, and participants were free to withdraw at any time.

We designed our 41-item survey, which is provided in the (S1 File), based on an extensive review of the literature. Measures of interest included the following:

■ *Program director-resident relationship quality*. We used the seven-item Leader-Member Exchange tool (LMX-7) to assess the quality of relationship between program director and resident. The Cronbach’s alpha of this scale ranges between 0.85 to 0.93 [24, 29]. The items were scored on a five-point Likert-type scale, with varying responses to each question ranging from 1 (left) to 5 (right). Responses on the left, such as rarely, not a bit, not at all, none, extremely ineffective, and strongly disagree, indicate a low-quality dyadic relationship, while responses on the right, such as very often, a great deal, fully very high, strongly agree, and extremely ineffective, indicate a high-quality dyadic relationship. The total score can range from 7 to 35, and the higher score reflects higher quality of program director-resident relationship.
■ *Perceived departmental support*. We used the eight-item Perceived Organizational Support questionnaire (POS-8) to measure the level of perceived departmental support by residents [30]. The Cronbach’s alpha of this scale is 0.80. The items were scored on a seven-point Likert-type scale. Participant responses were averaged to create an overall perceived organizational support score ranging from 0 to 6. Higher scores indicate that respondents perceived their organization to be more supportive. The questions ask residents about the extent to which they agree or disagree with statements regarding different aspects of departmental support. Participants were told that these items might represent possible opinions they may have about their department and participants were asked to indicate their level or agreement or disagreement with the items using the seven-item Likert scale. Sample items include: “The department fails to appreciate any extra effort from me” and “The department values my contribution to its well-being”.
■ *Burnout*. To measure burnout among residents, we used the single-item Maslach Burnout Inventory tool (MBI-2), which is a shortened version of the original 22-item scale. The MBI-2 tool was designed to measure emotional exhaustion (EE) and depersonalization (DP) using one item for each sub-scale. These 2 items remain the property of Mind Garden Inc (which holds the copyright of the MBI) and were used with the appropriate license. They were also used in previous studies and showed the highest correlation with overall EE or DP score on the original MBI-22 tool [31, 32]. The items were scored on a seven-point Likert-type scale as follows: 0 = never, 1 = few times per year, 2 = once a month, 3 = a few times per month, 4 = once a week, 5 = a few times per week, 6 = every day. Percentage of participants answering ≥ 4 is consistent with high EE and/or DP. Burnout is characterized by high score on EE or DP. Residents were asked about the extent to which they agree with the statements that ask about: “I feel burned out from my work” and “I have become more callous toward people since I took this job”.
■ *Engagement*. To measure residents’ engagement, we used the Utrecht Work Engagement Scale (UWES-9), a shortened version of the original 24-item scale. Items cover three aspects of the work engagement concept: vigor, dedication, and absorption. The three factors of the UWES-9 were highly correlated, and a total score could be used for practical purposes. Reported Cronbach’s alpha of total scale (0.90) was within acceptable ranges [33, 34]. The items were scored on a seven-point Likert-type scale as follows: 0 = never, 1 = few times per year, 2 = once a month, 3 = a few times per month, 4 = once a week, 5 = a few times per week, 6 = every day. Although the nine-items UWES scale consists of three sub-scales, however, further confirmatory analysis was done for this scale, and no clear three-factor structure was reported [35]. Hence, a total score was computed, and a higher score indicate that residents were more engaged at work. Residents were asked about the extent to which they agree or disagree with the statements that ask about their different feelings at work. Sample items include: “At my work, I feel bursting with energy” and “My job inspires me”.
■ *Quality of patient care (QOC)*. Residents’ self-reported quality of care was measured using 10-item QOC questionnaire, adapted from two research studies with appropriate permissions. Items cover three distinct predictors: suboptimal patient care practices, medical errors, and suboptimal attitudes towards patients. The items were scored on a five-point Likert-type scale as follows: 1 = never, 2 = rarely, 3 = sometimes, 4 = fairly often, 5 = very often. Internal consistency for the suboptimal practices and medical errors sub-scales was estimated by Cronbach’s alpha, and reliability coefficients for the two subscales were 0.75 and 0.60 consecutively [36]. The two items on suboptimal attitudes subscale were extracted from the 6-item “interpersonal disengagement” subscale [37], and their internal consistency will be assessed using Cronbach’s alpha. Participant responses were averaged on each subscale but not summarized to give a composite score on the full scale. The measures on each subscale are inverse, and higher scores on each quality predictor means lower quality of care. Residents were asked about the frequency of engaging in common suboptimal patient care practices, frequency of medical errors, and frequency of engaging in sub-optimal attitudes with the patient during the last 3 months. Sample items include: “work while impaired by fatigue”, “feel less empathetic with your patients”.
■ *Other variables*. The following sociodemographic data were recorded: age, gender, postgraduate year level (PGY), specialty, and number of working hours.

We analyzed data using IBM SPSS Statistics 24 and summarized continuous variables using mean ± SD, and categorical variables using frequency. We calculated Cronbach’s alpha to assess the internal consistency of the suboptimal attitudes sub-scale. Using SPSS macro-PROCESS by Preacher and Hayes [38], we used ordinary least squares regression to conduct mediation and moderated mediation analyses, to test the proposed research model previously shown in Fig 1.

Parallel mediation analyses (Model 4) were conducted to examine mediation effects of emotional exhaustion (EE), depersonalization (DP) and engagement (ENG) on the association between program director-resident relationship quality and quality of care dimensions. The macro allows calculating and testing the direct effect (regression controlled for the mediator), the total effect (regression without including the mediator), and the indirect effect.

Next, we conducted moderated mediation analyses (Model 7) to investigate whether perceived departmental support moderates the indirect effect of program director-resident relationship quality on each of the three quality of care dimensions through each of the wellbeing dimensions (EE, DP; and ENG). The macro allows assessing whether the indirect effect is conditional on the values of another variable, called a moderator.

The mediation and moderated mediation analyses were based on bootstrapping (5000 bootstrap samples) using 95% confidence intervals, and significance was set at *p* value of .05. Mediation was significant when the 95% CI did not include 0. Moderated mediation was significant if the 95% CI of the Index of Moderated Mediation did not include 0.

This study was approved by the Institutional Review Board of the participating study site (protocol SBS-2020-0104, approved July 5, 2020).

### Post hoc statistical power analysis

Post hoc power analyses were conducted using the G*Power software. The sample size of 95 was used for the statistical power analyses. The recommended effect sizes used for this assessment were as follows: small (f^2^ = .02), medium (f^2^ = .15), and large (f^2^ = .35) as per Cohen’s [39]. The alpha level used for this analysis was 0.05.

For each mediation pathway analysis (e.g., LMX → EE → QOC 1), a 2-predictor variable equation was used as a baseline. Post hoc analyses revealed the statistical power for this study was .21 for detecting a small effect, whereas the power exceeded .90 for the detection of a medium to large effect size. Thus, there was more than adequate power (i.e., power ≥ .80) at the medium to large effect size level, but less than adequate statistical power at the small effect size level.

For each moderated mediation pathway analysis (e.g., LMX*POS → EE → QOC 1), a 3-predictor variable equation was used as a baseline for each moderated mediation pathway. Post hoc analyses revealed the statistical power for this study was .02 for detecting a small effect, whereas the power exceeded .88 for the detection of a medium to large effect size. Thus, there was more than adequate power (i.e., power ≥ .80) at the medium to large effect size level, but less than adequate statistical power at the small effect size level.

## Results

Of 332 residents, 95(28.6%) responded. Descriptive statistics for study constructs by demographic characteristics are shown in Table 1. The mean score on the LMX-7 tool was 23.41 (± 6.02), consistent with moderate quality of relationship between residents and program directors, and the mean score on the POS-8 tool was 3.67 (± 1.36) consistent with low level of perceived departmental support by residents. In this sample, 34 (35.79%) residents reported a mean score of 4.22 on the POS tool within the -1 SD group (consistent with high POS), while 24 (25.26%) reported a mean score of 4.22 on the POS tool within the +1 SD group (consistent with high POS). The difference between both mean scores was significant (*p* < .001).

**Table 1:** Descriptive statistics for study constructs by demographic characteristics.

Demographic attribute		*N*	*%*	LMX	POS	EE^c^	DP^d^	Burnout^e^	ENG	QOC-1	QOC-2	QOC-3
				*M (SD)*	*M (SD)*	*N* (%)	*N* (%)	*N* (%)	*M (SD)*	*M (SD)*	*M (SD)*	*M (SD)*
**Gender**	Male	38	40	23.16 (5.61)	3.63 (1.28)	17 (44.7)	14 (36.8)	22 (57.89)	4.12 (1.18)	2.39 (.54)	1.67 (.47)	2.35 (.89)
	Female	57	60	23.57 (6.32)	3.69 (1.43)	28 (49.1)	20 (35.1)	32 (56.14)	4.30 (1.14)	2.47 (.58)	1.56 (.46)	2.62 (1.02)
**PGY level**	1	19	20	21.89 (6.38)	3.91 (1.26)	11 (57.9)	6 (31.6)	12 (63.16)	4.47 (.89)	2.58 (.48)	1.65 (.46)	2.24 (.81)
	2	21	22	25.00 (6.14)	3.92 (1.37)	13 (61.9)	8 (38.1)	16 (76.19)	4.52 (.98)	2.38 (.46)	1.60 (.43)	2.17 (.68)
	3	28	29.5	22.18 (5.62)	3.27 (1.30)	12 (42.9)	10 (35.7)	12 (42.86)	4.00 (1.03)	2.54 (.65)	1.69 (.57)	3.00 (.87)
	4	21	22.1	25.14 (6.10)	3.84 (1.56)	8 (38.1)	10 (47.6)	10 (47.62)	4.00 (1.54)	2.37 (.59)	1.46 (.36)	2.71 (1.24)
	>4	6	6.31	22.33 (4.72)	3.23 (1.03)	2 (33.33)	0 (0)	1 (16.67)	4.31 (1.45)	1.94 (.49)	1.56 (.40)	1.67 (.52)
**Working hrs**	>80 hrs	16	16.8	25.62 (5.86)	3.85 (1.24)	8 (50)	6 (37.5)	9 (56.25)	4.46 (1.07)	2.33 (.52)	1.60 (.53)	2.38 (.74)
	71–80 hrs	36	37.9	21.78 (6.39)	3.36 (1.33)	22 (61.1)	14 (38.9)	25 (69.44)	4.21 (1.03)	2.56 (.59)	1.61 (.45)	2.46 (.95)
	61–70 hrs	18	18.9	23.00 (4.96)	3.80 (1.23)	7 (38.9)	7 (38.9)	10 (55.56)	3.81 (1.35)	2.48 (.55)	1.59 (.35)	2.72 (1.19)
	51–60 hrs	18	18.9	24.28 (5.85)	3.86 (1.49)	8 (44.4)	5 (27.8)	8 (44.44)	4.37 (1.00)	2.43 (.45)	1.74 (.53)	2.69 (.81)
	41–50 hrs	6	6.3	25.67 (6.92)	3.92 (2.02)	0 (0)	0 (0)	2 (33.33)	4.67 (1.82)	2.00 (.67)	1.28 (.33)	2.17 (1.51)
	≤ 40 hrs	1	1.1	25.00	4.13	0 (0)	0 (0)	0 (0)	3.56	1.33	1.00	2.00
**Specialty**	Surgical^a^	45	47.4	22.71 (5.33)	3.59 (1.29)	22 (48.9)	15 (33.3)	26 (57.78)	4.07 (1.17)	2.47 (.54)	1.58 (.43)	2.46 (.93)
	Non-Surgical^b^	50	52.6	24.04 (6.57)	3.73 (1.43)	23 (46)	19 (38)	27 (54)	4.37 (1.13)	2.41 (.60)	1.63 (.50)	2.57 (1.02)

N = 95.

^a^Surgical specialties included anesthesiology, obstetrics & gynecology, general surgery, and all surgical subspecialties.

^b^Non-Surgical specialties included anatomic pathology, laboratory medicine, dermatology, ophthalmology, diagnostic radiology, radiation oncology, emergency medicine, family medicine, internal medicine, neurology, pediatrics and psychiatry.

^c^High score (at least weekly) on the emotional exhaustion.

^d^High score (at least weekly) on the depersonalization.

^e^High score (at least weekly) on the emotional exhaustion or depersonalization scale.

LMX, Program director-resident relationship quality; POS, Perceived departmental support; EE, Emotional exhaustion; DP, Depersonalization; ENG, Engagement; QOC 1, Suboptimal patient care management practices; QOC 2, Medical errors; QOC 3, Suboptimal attitudes towards patients.

Among respondents, 54(56.8%) reported either a high EE (47.4%) or a high DP (35.8%) on the MBI-2 tool, meeting the criteria for burnout. The mean score on the UWES-9 tool was 4.23 (± 1.15), consistent with moderate level of engagement among respondents. Residents reported committing medical errors less frequently (mean 1.60 ± .57) than engaging in suboptimal patient care management practices (mean 2.44 ± .46) and suboptimal attitudes with patients (mean 2.51 ± .97). The suboptimal attitudes subscale had a high level of internal consistency as determined by a Cronbach’s alpha of .88.

In parallel mediation analyses, we found that program director-resident relationship quality had significant total effect on frequency of engaging in suboptimal patient care management practices (*b* = -.0257, 95% CI -.0444, -.0070, *p <* .05), and frequency of engaging in suboptimal attitudes towards patients (*b* = -.0434, 95% CI -.0755, -.0114, *p <* .05), the direct effects were not (*p* > .05) and indirect effects (IEs) were present. As shown in Table 2, we also found that program director-resident relationship quality had significant IE on suboptimal patient care management practices via EE in the expected direction (*b* = -.0076, 95% CI -.0173, -.0005, *p <* .05), whereby the CI suggests non-zero values. However, the effect size was small (*ab*_*ps*_ = -.0134, *ab*_*cs*_ = -.0809) as per Cohen’s [39]. Recalling the post hoc power analysis revealed low power for finding small effects in each mediation pathway, we believe that this finding would have a stronger level of significance given more statistical power. In addition, program director-resident relationship quality had significant IE on suboptimal attitudes towards patients via DP (*b* = -.0196, 95% CI -.0387, -.0049, *p* < .05) and ENG (*b* = -.0177, 95% CI -.0361, -.0039, *p* < .05). The respective partially standardized effect sizes were *ab*_*ps*_ = -.0202, -.0182. The respective completely standardized indirect effects for DP and ENG were *ab*_*cs*_ = -.1214, -.1095, and approaching a moderate effect size as per Cohen’s [39]. The power to detect an effect of this size in this mediation pathway analysis was determined to be > .90. Indirect effects are listed in Table 2.

**Table 2:** Indirect effects for the paths on the parallel mediation models (PMMs).

		Indirect effects of LMX on QOC	
		*b (SE)*	*95% CI (LL*, *UL)*
PMM 1: LMX → EE, DP, ENG → QOC 1	**LMX → EE → QOC 1**	**-0.0076 (.0043)**	**(-.0173, -.0005)**
	LMX **→** DP **→** QOC 1	-.0038 (.0035)	(-.0118, .0020)
	LMX **→** ENG **→** QOC 1	.0017 (.0042)	(-.0068, .0100)
PMM 2: LMX → EE, DP, ENG → QOC 2	LMX **→** EE **→** QOC 2	-.0034 (.0032)	-.0106, .0022
	LMX **→** DP **→** QOC 2	-.0025 (.0029)	-.0087, .0028
	LMX **→** ENG **→** QOC 2	-.0010 (.0029)	(-.0074, .0044)
PMM 3: LMX → EE, DP, ENG → QOC 3	LMX **→** EE **→** QOC 3	-.0055 (.0062)	(0177, .0077)
	**LMX → DP → QOC 3**	**-.0196 (.0084)**	**(-.0378, -.0049)**
	**LMX → ENG → QOC 3**	**-.0177 (.0082)**	**(-.0361, -.0039)**

N = 95. b represents unstandardized regression coefficients with ordinary least squares (OLS) regression method; Paths in bold indicate statistically significant indirect effects.

SE represents standardized effects. Bootstrapped 95% CIs. LL—Lower Limit, UL—Upper Limit. LMX, Program director-resident relationship quality; EE, Emotional Exhaustion; DP, Depersonalization; ENG, Engagement; QOC 1, Suboptimal patient care management practices; QOC 2, Medical errors; QOC 3, Sub-optimal attitudes towards patients.

**Fig 2:**
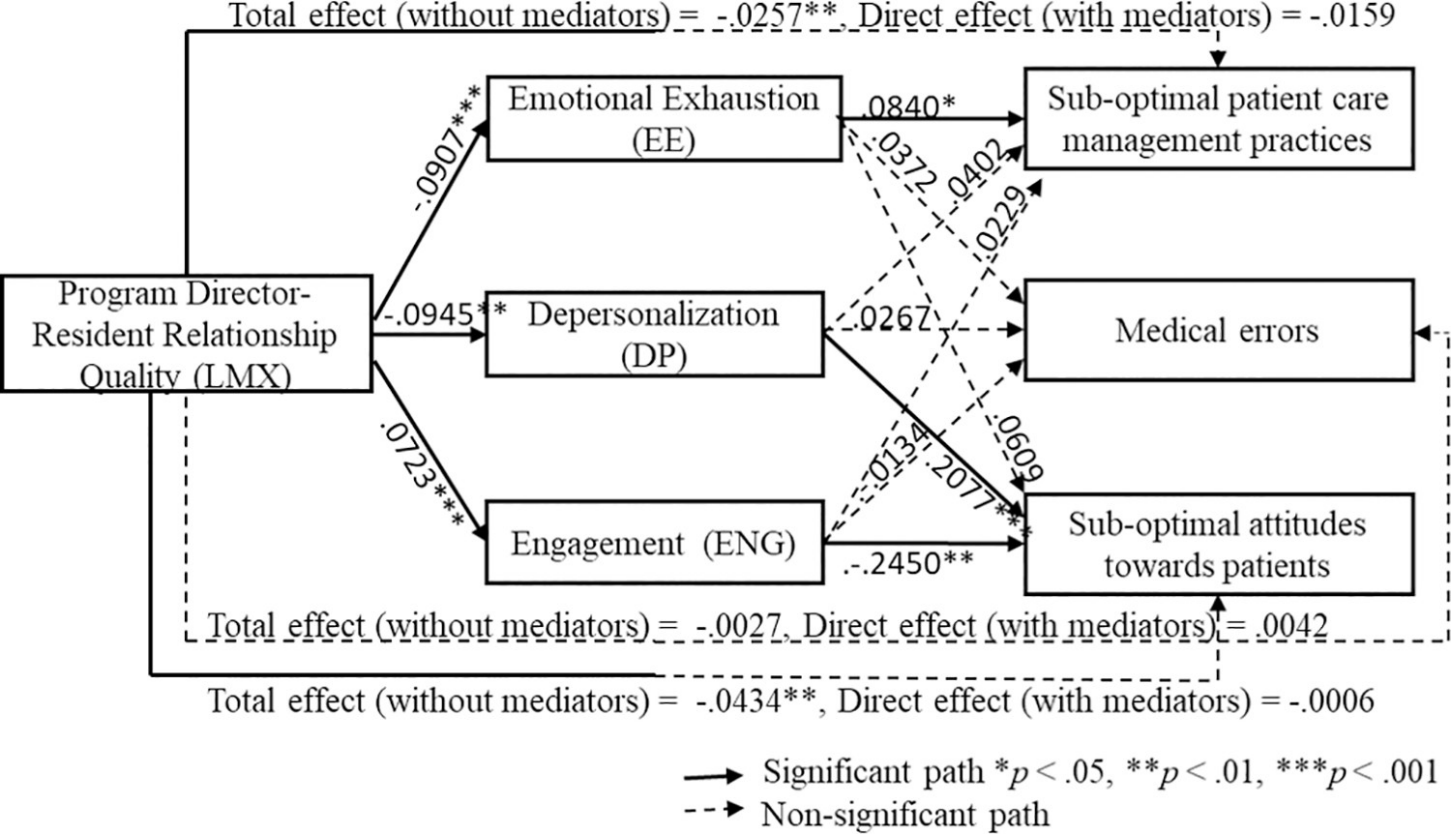
Testing the parallel mediation model for direct effects and total effects.

Through further exploration of the direct effects within these parallel mediation analyses, we found that program director-resident relationship quality had a significant negative effect on EE and DP (*p* < .001 and *p* = .0028 respectively), and significant positive effect on ENG (*p* < .001). In addition, we found that EE had a significant positive effect on engaging in suboptimal patient care management practices (*b* = .0840, 95% CI .0035, .1645, *p* = .04), DP had a significant positive effect on engaging in suboptimal attitudes towards patients (*b* = .2077, 95% CI .1062, .3092, *p* < .001), and ENG had significant negative effect on engaging in suboptimal attitudes towards patients (*b* = -.2450, 95% CI -.3997, -.0904, *p* < .01). Total and direct effects are represented in Fig 2.

We conducted moderated mediation analyses to test whether the IEs observed earlier were significantly different in residents with low and high levels of perceived departmental support. As shown in Table 3, we found that the conditional indirect effect of program director-resident relationship quality on suboptimal patient care practices through EE was non-significant (index = .0021, 95% CI -.0016, .0058). We also found that the partially standardized and completely standardized effect sizes were small at different levels of the moderator (*ab*_*ps*_ < .02, *ab*_*cs*_ < .02). In moderated mediation analysis, standardized effect size measures are obtained by standardizing the conditional indirect effects of X on Y at various values of the moderator. Similarly, we found that the conditional indirect effects of program director-resident relationship quality on suboptimal attitudes towards patients through DP (index = .0003, 95% CI -.0117, .0121) and ENG (index = -.0063, 95% CI -.0132, .0007) were also non-significant. The respective partially standardized and completely standardized effect sizes were also small (*ab*_*ps*_ < .02, *ab*_*cs*_ < .02). Additionally, the post hoc power analysis previously revealed low power for finding small effects in each moderated mediation pathway.

**Table 3:** Moderated indirect effects.

Conditional indirect effect (through EE) on QOC1: Suboptimal patient care management practices		***b (SE)***	***95% CI (LL*, *UL)***
	Perceived departmental support (- 1 SD)	-.0123 (.0066)	(-.0270, -.0013)
	Perceived departmental support (+ 1 SD)	-.0067 (.0058)	(-.0205, .0027)
		***Index***	** *95% CI (LL*, *UL)***
	Index of moderated mediation	.0021 (.0018)	(-.0016, .0058)
Conditional indirect effect (through DP) on QOC 3: Suboptimal attitudes towards patients		***b (SE)***	***95% CI (LL*, *UL)***
	Perceived departmental support (- 1 SD)	-.0086 (.0173)	(-.0431, .0271)
	Perceived departmental support (+ 1 SD)	-.0077 (.0198)	(-.0461, .0335)
	* *	***Index***	**(-.0343, .0164)**
	Index of moderated mediation	.0003 (.0061)	(-.0117, .0121)
Conditional indirect effect (through ENG) through QOC 3: Suboptimal attitudes towards patients		***b (SE)***	***95% CI (LL*, *UL)***
	Perceived departmental support (- 1 SD)	.0087 (.0125)	(-.0161, .0340)
	Perceived departmental support (+ 1 SD)	-.0084 (.0127)	(-.0343, .0164)
		***Index***	** *95% CI (LL*, *UL)***
	Index of moderated mediation	-.0063 (.0036)	(-.0132, .0007)

N = 95. b represents unstandardized regression coefficients with ordinary least squares (OLS) regression method. SE represents standardized effects. Bootstrapped 95% CIs. LL—Lower Limit, UL—Upper Limit. LMX, Program director-resident relationship quality; EE, Emotional Exhaustion; DP, Depersonalization; ENG, Engagement; QOC 1, Suboptimal patient care management practices; QOC 2, Medical errors; QOC 3, Sub-optimal attitudes towards patients.

## Discussion

We found that burnout subcomponents (exhaustion and depersonalization) were directly associated with residents’ perceptions of providing poorer quality of care, while engagement was directly associated with residents’ perceptions of providing better quality of care. Although we did not find that program director-resident relationship quality was directly related to any of the quality-of-care dimensions, it was negatively associated with emotional exhaustion, depersonalization and was positively associated with engagement, thereby indirectly influencing quality of care. In addition, we found that the observed indirect effects were not significantly different in residents with low and high levels of perceived departmental support.

Our findings were similar to previous studies, which suggested a relationship between burnout subcomponents and quality of care [5, 40]. Earlier evidence on residents’ engagement and quality of care is limited to two studies conducted by Prins *el al*. and Loerbrocks *et al*. [6, 7], and our findings on engagement are consistent with those studies. Depersonalization and engagement showed strong ties with suboptimal attitudes, while emotional exhaustion was associated with suboptimal patient care. Hence, depersonalization and engagement might have more immediate effects on interactions with patients, while emotional exhaustion that depletes the individual’s energy impairs the quality of patient care management practices.

We also found that program director-resident relationship quality was positively associated with residents’ engagement in the workplace. Previous findings could explain the mechanism behind these associations; employees in high-quality relationships with their leaders were more enthusiastic and confident about their abilities to execute and succeed. Such beliefs were significant predictors of work engagement, which improved employees’ execution of tasks [41, 42]. Hence, a high-quality relationship between a program director and resident can act as a vital job resource for residents and help create an environment that is more supportive of their needs and values. According to Schaufeli and Bakker [34], job resources have been identified as the main drivers of work enjoyment, motivation, and work engagement, through promoting psychological safety. This, in turn, leads to increased wellbeing and positive organizational outcomes. Examples of these job resources are social support from colleagues, high-quality relationship with the supervisor, and performance feedback [34].

In the present study, we also found that a higher quality of program director-resident relationship was associated with lower residents’ burnout subcomponents. This follows the line of Tepper [43], who stated that the low quality of leader-member relationships is one of the most common sources of stress in organizations. Similarly, findings in the literature reported that employees with low-quality relationships with leaders exhibit low morale, perceive limited leadership support, and high-stress levels [44, 45]. According to the job-demands resource model, supportive relationships with leaders provide followers with job resources (e.g., performance feedback, trust) to promote engagement, and monitor their quantitative and qualitative work demands (e.g., work overload) to decrease the risk of burning-out [46]. Hence, program director’s support might act as an ’antidote’ to work strain by promoting job resources and monitoring job demands. This could help residents cope with their stressful work environment and reduce their risk of burning out.

Furthermore, our results show that program director-resident relationship quality was significantly indirectly associated with a higher quality of care by reducing residents’ emotional exhaustion and depersonalization and promoting their workplace engagement. More specifically, high quality of program director-resident relationship was indirectly significantly associated with lower frequency of sub-optimal patient care practices and attitudes towards patients. The mechanism behind the observed association could be explained by the leader-member exchange theory [17]. Residents who feel supported by their program directors feel more psychologically safe, more capable of dealing with job strains, more committed and motivated to perform well, and avoid adverse clinical health outcomes. Based on the present study results, it is warranted to extend the research investigation further in order to examine whether the improvement in quality of care through the indirect effect of program director-resident relationship quality will be affected by other factors in the residents’ work environment.

Although there was a significant difference between the mean scores on the POS tool within ± 1 SD, yet we did not find that the observed indirect effects were significantly different in residents with low and high levels of perceived departmental support. This implies that perceived departmental support did not have a dominant role over program director-resident relationship quality and did not moderate the indirect effects. This insignificant moderation effect is however constrained by the low statistical power obtained from the post hoc analysis. Our study is nevertheless exploratory in nature, and its findings warrant further investigations about the role that departmental support and program director leadership can jointly play in supporting residents’ wellbeing and their clinical performance. Although there is much work to be done on this subject, our study findings may suggest that even when the department does not care enough for the residents, those with a high-quality relationship with their program directors are very likely to experience reduced burnout and higher levels of engagement and less likely to perform poorly in their patient care practices. Conversely, a poor relationship with the program director will hardly enhance residents’ engagement and reduce their burnout, even if the departments offer all types of benefits. This may be related to the nature of the medical education programs where residency programs are conveyed and eventually implemented by program directors, with whom the residents must deal face to face for all residency matters. Hence, program directors may play an essential and irreplaceable role in promoting residents’ wellbeing and reducing the suboptimal quality of care.

Many residency programs in Lebanon have guidelines and curricula in place that address and promote residents’ wellbeing. These entail a policy about resident impairment which permits the diagnosis of burnout and depression, provides opportunity for treatment as well as the possibility of returning back to work. The goals of this policy are to protect patients against any potential risks of suboptimal care provided by impaired resident as well as compassionately helping the impaired resident recover and return back to duty. In addition, residency program curricula incorporate mental health lecture series as well as workshops that aim to provide residents with techniques to shift their attention to effective ways of coping with uncertainties and heavy job demands.

However, the results of this study could send important messages to healthcare executives in academic medical centers. One message is the significant role of program directors in promoting residents’ wellbeing and improving their performance. Because the program director might play a pivotal role in affecting residents’ engagement levels as well as burnout and performance in terms of clinical outcomes, academic medical centers should consider conducting programs for training program directors to improve their social exchange relationships with residents and improve their supervisory and interpersonal skills. It might also be appropriate to longitudinally evaluate the quality of the program director-resident relationship as one of the residency program performance indicators. Similar to previous studies, our results present empirical evidence on the relationship between residents’ wellbeing and quality of care. This could provide an additional evidence-based recommendation for medical centers to adding a fourth dimension, which is improving physician’s wellbeing, to their triple aim of better care, better health, and lower costs.

## Limitations

Our study had several limitations. First, the timing of data collection was not optimal as it occurred during the COVID-19 pandemic, and during unseen economic hardship and financial threats in Lebanon. Hence, residents might have had strong aversion to participate in any research study, and this could have reduced our survey response rate. Second, such challenging circumstances could have affected residents’ stress, engagement, and performance levels. Third, obtaining data from a single center raises concerns regarding generalizability of the findings. Fourth, the modest sample size in the present study (*N* = 95) may have played a role in limiting the significance of some of the statistical analyses conducted and particularly for the conditional indirect effects. Hence, this is a constraint that needs to be addressed through replication and additional research. Fifth, we have used the shortened two-question burnout questionnaire instead of the original Maslach Burnout Inventory tool for practical reasons. Finally, the measurement of quality of care was based on self-reported answers and not on an audit of medical records due to the anonymous nature of the study. Hence, the extent to which perceived medical errors reflect the frequency of medical errors and whether these perceived medical errors affect patient outcomes could not be determined. Despite this limitation, 53% of self-perceived errors have been found to affect actual patient outcomes in some studies [47].

## Conclusion

Our study adds a new dimension to the body of literature suggesting that program director leadership plays an important role in promoting residents’ wellbeing and improving their clinical performance. Our results show that residents in high-quality relationships with their program directors might be less likely to experience burnout, more likely to be engaged, and less likely to perform poorly in their patient care practices. Further research studies are needed to further confirm our findings. This may provide additional insight into the crucial role that program directors should play in creating more favorable and productive work environments, and further support our policy/programmatic recommendations.

## Supporting information

S1 File Supplementary material. The 41-item questionnaire and corresponding psychometric properties.
(PDF)
