# Supplementary material for: The mediating role of residents’ wellbeing between program leadership and quality of care: A cross-sectional study
Source: PLoS One. 2021 Nov 8;16(11):e0259800. doi: 10.1371/journal.pone.0259800 (PMC8575271; doi:10.1371/journal.pone.0259800)
Supplement: S1 File — The 41-item questionnaire and corresponding psychometric properties. (PDF) [file pone.0259800.s001.pdf]

Supplemental Digital Appendix 1. The 41-Item Questionnaire and Corresponding Psychometric Properties

Demographic and Professional Chracteristics
Age
Gender
Post Graduate Year Level
Specialty
Average number of working hours per week

Instrument	Construct	Psychometric properties	Item code	Item
Leader Member Exchange (LMX-7)	Program director-resident relationship quality (LMX)	Cronbach's alpha = 0.85 to 0.93	LMX 1	Do you know where you stand with your program director [and] do you usually know how satisfied your program director is with what you do?
			LMX 2	How well does your program director understand your job problems and needs?
			LMX 3	How well does your program director recognize your potential?
			LMX 4	Regardless of how much formal authority your program director has built into his or her position, what are the chances that your program director would use his or her power to help you solve problems in your work?
			LMX 5	Again, regardless of the amount of formal authority your program director has, what are the chances that he or she would “bail you out” at his or her expense?
			LMX 6	I have enough confidence in my program director that I would defend and justify his or her decision if he or she were not present to do so.
			LMX 7	How would you characterize your working relationship with your program director?
Perceived Organizational Support (POS-8)	Perceived departmental support (PDS)	Cronbach's alpha = 0.80	PDS-1	The department values my contribution to its well-being.

			PDS-2	The department fails to appreciate any extra effort from me.
			PDS-3	The department would ignore any complaint from me.
			PDS-4	The department really cares about my well-being.
			PDS-5	Even if I did the best job possible, the department would fail to notice.
			PDS-6	The department cares about my general satisfaction at work.
			PDS-7	The department shows very little concern for me.
			PDS-8	The department takes pride in my accomplishments at work.
Maslach Single Items Burnout Survey (MBI-2)	Emotional Exhaustion (EE)	Spearman's r with parent sub-scale = 0.89, $p < .0001$	EE	I feel burned out from my work
	Depersonalization (DP)	Spearman's r with parent sub-scale = 0.81, $p < .0001$	DP	I have become more callous toward people since I took this job
Utrecht Work Engagement Scale (UWES-9)	Engagement (ENG)	Cronbach's alpha = 0.90	ENG-1	At my work, I feel bursting with energy
			ENG-2	At my job, I feel strong and vigorous
			ENG-3	When I get up in the morning, I feel like going to work
			ENG-4	I am enthusiastic about my job
			ENG-5	My job inspires me
			ENG-6	I am proud of the work that I do
			ENG-7	I feel happy when I am working intensely
			ENG-8	I am immersed in my work
			ENG-9	I get carried away when I'm working
Quality of Patient Care (QOC-10)	Frequency of engaging in common suboptimal patient care practices (QOC-1)	Cronbach's alpha = 0.75	During your last 3 months, how often did you	
			QOC-1.1	Work while impaired by fatigue
			QOC-1.2	Forget to transmit important information during sign-out

		QOC-1.3	Report information that you were unsure of
		QOC-1.4	Write information in a patient’s chart that you were unsure of
		QOC-1.5	Make up information to report to your superior
Frequency of engaging in medical errors (QOC-2)	Cronbach's alpha = 0.60	During your last 3 months, how often did you make the following avoidable medical errors, which were not due to lack of medical knowledge?	
		QOC-2.1	Cognitive (wrong test, wrong diagnosis, wrong treatment)
		QOC-2.2	Technical (procedural error e.g. pneumothorax)
		QOC-2.3	Administrative errors (patient record error, patient identification error, follow up errors, communication failure during transitions of care)
Frequency of engaging in sub-optimal attitudes with the patient (QOC-3)	Cronbach's alpha = 0.88	During your last 3 months, how often did you...?	
		QOC-3.1	Feel less empathetic with your patients
		QOC-3.2	Feel less interested to talking with your patients
